# Oxidative Stress Evaluation in Patients Treated with Orthodontic Self-ligating Multibracket Appliances: An *in Vivo* Case-Control Study

**DOI:** 10.2174/1874210601711010257

**Published:** 2017-06-30

**Authors:** Marco Portelli, Angela Militi, Gabriele Cervino, Floriana Lauritano, Sergio Sambataro, Alberto Mainardi, Riccardo Nucera

**Affiliations:** 1 Department of Biomedical, Dental Science and Morphological and Functional Images, Dental School, University of Messina, Messina, Italy; 2 Private Practice, Center Orthodontics and Gnathology (COS), Catania, Italy; 3 CIR Dental School of Turin, University of Turin, Turin, Italy

**Keywords:** Oxidative stress, Multibrackets orthodontic appliance, Salivary antioxidant test, Extra-cellular components, Apoptosis

## Abstract

**Objective:**

Oxidative stress is a pathologic event induced by a prevalence of oxidant agents on the antioxidant ones, with a consequent alteration of oxide-reducing balance.

**Introduction:**

Freeradicals produce damages both in cellular and extra-cellular components; phospholipid membranes, proteins, mitochondrial and nuclear DNA, are the target of the oxidative stress, that can finally cause cellular death due to apoptosis.

**Materials & Methods:**

Orthodontic appliances such as brackets, wires, resins and soldering have some components that can be considered as potential allergen, carcinogenic, cytotoxic and gene mutation factors. The primary aim of this research is to evaluate oxidative stress in the saliva of patients treated with multibracket self-ligating vestibular orthodontic appliances; the secondary purpose is to investigate the influence of orthodontic multibracket therapy on oral hygiene and the consequent effect on oxidative stress. Salivary specimens has been collected in a sample of 23 patients were enrolled (12 Female, 11 Male) between 12 and 16 years of age (mean age 14.2). For each patient has been collected a salivary specimen at the following time points; before orthodontic bonding (T1), five weeks (T2) and ten weeks (T3) after orthodontic appliance bonding.

**Results:**

Samples has been analysed with a photometer due to SAT Test (Salivary Antioxidant Test). Data obtained show a mean of 2971 mEq/l of anti-oxidant agents before orthodontic treatment, and after five weeks from the bonding the mean was decreased to 2909 mEq/l, instead at ten weeks was increased to 3332 mEq/l. Repeated measures ANOVA did not reveal statistically significant differences between the time points (*P* = 0.1697). The study did not reveal any correlation between the level of dental hygiene and that of oxidative stress (Pearson Correlation Coefficient R = 0).

**Conclusion:**

Orthodontic treatment with multibrackets vestibular metallic appliance seems to be not able to affect oxidative stress during the first ten weeks of therapy.

## INTRODUCTION

In recent years, clinical and experimental medicine has shown great interest in oxidative stress as a trigger event for several human diseases. Oxidative stress is a pathologic asymptomatic event, highly detrimental to tissues and cells, induced by a prevalence of oxidizing agents over antioxidant ones, with a consequent alteration of oxidation/reduction balance. A free radical is a highly reactive chemical species, characterized by an unpaired electron in its external orbital; this electron allows linkage to other radicals or the snatching of one electron from adjacent molecules [1]. This event produces the oxidation of the adjacent molecule with important damage to its structure and functions: free radicals can produce detrimental effects on cellular proteins, creating modified amino acids, and on nucleic acids, especially the mitochondrial ones, creating alternative nitrogen bases. These can produce a chain-reaction effect. In human beings, free radicals are generated by exposure to UV, X- and gamma rays and can also be produced by leukocytes and macrophages during inflammatory processes. Free radicals are characterized by a short lifetime; however, some species, such as molecular oxygen, persist for a long time. From a biological and pathological point of view, the most important free radicals are the reactive oxygen species (ROS); the ratio between ROS and antioxidant molecules defines the oxide-reducing balance, which is one of the most important factors, after genetic ones, in the determination of lifespan and quality of life [2]. Among the free radicals generated in the human organism, the following must be considered:

Superoxide Anion (O_2_*) is generated under both physiological and pathological conditions by neutrophils and macrophages [3].

**Hydrogen Peroxide (H_2_O_2_)** is considered a relatively weak free radical and is produced in all systems that generate superoxide anion.

**Hypo chloric Acid (HOCl)** can be neutralized to water (H_2_O) or metabolized by the myeloperoxidase enzyme and transformed into a strong oxidizing agent. This reaction is considered highly relevant in the inflammatory process, because the myeloperoxidase protein is one of the most represented proteins in the phagocytes [4].

Hydroxyl Radical (OH*) is considered the most active free radical. In competition with H_2_O_2_, it inactivates mitochondrial enzymes and causes direct damage to DNA.

Considering the short lifetime and high level of reactivity, the damage produced by a free radical is usually limited to the tissue in which the radical is developed. The most important cellular targets of oxidative stress are the phospholipid membranes [1]; the increase in hydro peroxides inside the cellular membrane produces modifications in its fluidity, trans-membrane enzyme activity, transport canals, membrane receptors, and other proteins [5]. Other important targets of free radicals are the proteins that represent the most plentiful cellular component; a simple modification of a single protein can produce an important effect on its functionality and biological activity [6]. Nuclear and mitochondrial DNA are two other important sites of oxidative stress; chain fracture, base hydrolysis, ATP depletion, and genetic mutation are the most common modifications induced. Finally, oxidative stress can produce cellular death due to apoptosis [7] free radicals, however, also contribute to physiological activity of the human organism such as immune system stimulation, blood pressure regulation, intra- and extracellular communication, and the synthesis of hormones such as catecholamine. Several factors, such as radiation, pollution, smoke, viruses, and bacteria, can modify oxido-reductive balance on the side of the oxidant species, producing oxidative stress; this is at the root of several human diseases, such as diabetes, atherosclerosis, hypertension, colitis, rheumatoid arthritis, *etc* [8, 9], and plays a fundamental role in aging [10]. Denham Harman proposed a therapy regarding aging; according to this therapy, aging is considered a consequence of the accumulation of detrimental effects produced by reactive oxygen species (ROS), and the capability of the organism to contrast these effects plays a fundamental role in life span [11]. Several diseases, such as Alzheimer’s [12], Parkinson’s, Huntington’s [13], and Steiner’s [14] seem to be related to mitochondrial defects induced by oxidative stress. The oxidative stress can be induced by X-Ray [15] and seems to be involved in the development of white spot lesions, enamel surface alterations, and dental caries, and the saliva can be considered the first barrier to oxidative stress; [16-21] the antioxidant capability of the saliva is essential for the maintenance of oral cavity balance and to avoid the development of local diseases. The primary aim of this research is to evaluate oxidative stress in the saliva of patients treated with multibracket self-ligating vestibular orthodontic appliances; the secondary purpose is to investigate the influence of orthodontic multibracket therapy on oral hygiene and the consequent effect on oxidative stress.

## MATERIALS AND METHODS

For the present study, 23 patients were enrolled (12 Female, 11 Male) between 12 and 16 years of age (mean age 14.2); vestibular self-ligating brackets (Smart Clip, 3M-Unitek) with MBT^TM^ prescription and Thermal Ni-Ti wires 0.014 Inch, Ortho-form II (Nitinol Heat Activated 3M-Unitek) were used. For each patient, approval was obtained to perform orthodontic therapy, and informed consent was given by a parent or guardian for the collection of salivary samples. The study has been approved by the Ethics Committee. The following inclusion criteria were used in the study:

- Permanent dentition
- Angle Class 1 malocclusion with moderate dental crowding
- Absence of systemic diseases that can influence oxidative stress
- No consumption of any type of medicine
- Availability to be enrolled in the study
- Absence of gingivitis or periodontal diseases

For each patient, a salivary sample was collected at different time points:

- Before the bonding of the multibracket appliance (T_0_)
- After 5 weeks (T_1_)
- After 10 weeks (T_2_)

For each patient, dental hygiene was evaluated before the collection of the salivary sample, using the Silness-Loe Plaque Index (PI) [22]. The PI is used together with the Gingival Index (GI), and is either used on all teeth (28 teeth, so wisdom teeth are excluded) or selected teeth. There is no substitution for any missing tooth. The PI is either used on all surfaces (M, O, D, L) or selected surfaces (M, O, L). This index measures the thickness of plaque on the gingival one third with the following scores:

0 = Absence of plaque.

1 = A film of plaque adhering to the free gingival margin and adjacent area of the tooth. The plaque may be seen in situ only after application of a disclosing solution or by using the probe on the tooth’s surface.

2 = Moderate accumulation of soft deposits within the gingival pocket or the tooth and gingival margin, which can be seen with the naked eye.

3 = Abundance of soft matter within the gingival pocket and/or on the tooth and gingival margin.

For the present study, a new photometer was used with an incorporated centrifuge (FRAS 5 EVOLVO : Free Radical Analytical System, H&D SPA, Italy), which utilizes D-ROMs, biological antioxidant potential (BAP) and the Salivary Antioxidant Test (SAT) for the evaluation of total oxidative stress. The D-ROMs test evaluates blood concentrations of metabolites and derivatives of oxygen (Reactive Oxygen Species, ROM) and in particular, hydro peroxide.

 The BAP (Biological Antioxidant Potential) test measures the efficiency of a plasmatic antioxidant barrier in terms of Fe-reduction activity. The SAT test is based on the capacity of a colored solution of Fe^3+^ to modify its coloring when Fe^3+^ has been reduced to Fe^2+^ due to the action of a reducing agent. A salivary sample was collected from fasting patients after at least 30 min from daily dental hygiene; in fact, foods and beverages such as coffee, tea, and fruit juice or the use of toothpaste can produce an alteration in anti-oxidant agents in the saliva. Before each sample, a small plastic glass and a square of cotton were weighed, taking note of the values registered. Patients were instructed to twirl the cotton in their mouth without biting, simulating chewing, in order to produce saliva. After 60 s, the small glass and cotton square were re-weighed, paying attention that the difference between the two measurements was in the range of 1.1 to 1.4 g.

 This range corresponds to the optimal salivary fluid expressed in ml/min, in order to obtain the maximum antioxidant effect and a constant amount of uric acid (principal antioxidant salivary agent). The cotton was wrung out by hand in order to collect the salivary sample in the small glass; in a cuvette containing the R_1_ reagent, 30 μl of the R_2_ reagent was added using a designated pipette and single-use tip. The cuvette was closed, shaken for 10 s and then inserted into the reading camera of the apparatus for the first reading. At a later time, the cuvette was removed and 10 μl of saliva was added to the solution containing R_1_ + R_2_; the cuvette was closed one more time, shaken, and inserted again into the reading camera. The reference values for the SAT test are expressed in mEq/L of antioxidant agents.

<1000 mEq/l - High deficiency

1000–1500 mEq/l - Optimal values

1500–2000 mEq/l - Normal values

2000–2500 mEq/l - Borderline values

>2500 mEq/l - Possible ongoing inflammation

For the study, 69 salivary samples have been collected; three for each patient enrolled; SAT test results were analyzed statistically by the analysis of variance (ANOVA) for repeated measures. PI values were analyzed by chi-square test. Statistical significance was set to α = 0.05. The software used for the statistical analysis was PRISM 6 (GraphPad Software, California, USA).

## RESULTS

The results of the SAT test are reported graphically in Fig. (**1**) and the descriptive statistic of the data is reported in Table **1**. Repeated measures ANOVA did not reveal statistically significant differences between the time points (*P* = 0.1697).

The results of the present study revealed a mean value of anti-oxidant agents in the saliva of patients before the treatment of 2954 mEq/l. Five weeks after bonding, the mean value reduced to 2903 mEq/l, instead 10 weeks after the beginning of the treatment, the mean concentration of antioxidant agents in the saliva increased to the extent of 3297 mEq/l. However statistical analysis did not reveal significant differences in the mean values of anti-oxidant agents among T_0_, T_1_, and T_2_. The distribution of the PI value in the study group, measured by the Silness-Loe gingival method, is reported in Table **2**. The distribution of the scores at the different time points is expressed graphically in Fig. (**2**). Statistical analysis performed by chi-square test did not reveal statistically significant differences between the groups (*P* > 0.05).

The results show that no patients had a PI value of 3 (Abondance of soft matter within the gingival pocket and/or on the tooth and gingival margin) at any time point. Patients with the scores of 0 maintained good oral hygiene at the following measurements: in the majority of patients with higher scores, the measurements at T_1_ and T_2_ were better; in some cases, the value remained the same. Considering worsening of dental hygiene during orthodontic treatment, the study did not reveal any correlation between the level of dental hygiene and that of oxidative stress (Pearson Correlation Coefficient R = 0). In the below table Table **3**, the correlation between PI scores and SAT results is illustrated.

## DISCUSSION

The primary aim of this research was to evaluate oxidative stress in the saliva of patients treated with multi-bracket vestibular orthodontic appliances; oxidative stress is produced due to the prevalence of free radicals over antioxidant agents. Considering their short life time, is very difficult to quantify free-radical concentration; to date, the only way to assess oxidative stress is to evaluate the consequent damage or to measure the concentration of substances produced to defend the organism [23]. In the literature, different studies are available [24-37] that describe the correlation between orthodontic therapy and the development of oxidant species. Some authors support that even if orthodontic brackets and wires are able to release harmful substances in the oral cavity, they are not cytotoxic; therefore, they do not produce oxidative stress [26, 27, 30]. Other recent studies [35-37] state that orthodontic brackets and wires produce an increase in free-radical concentration without producing acute toxicity, and stainless steel materials seem to be more biocompatible. The present research did not reveal statistically significant differences between the different Time Points. On the basis of the results obtained in this study, it is possible to state that multi-bracket, self-ligating orthodontic therapy with a vestibular appliance did not influence oral cavity oxidative stress in the first 10 weeks of treatment. The normal values of anti-oxidant agents in a healthy adult human subject are between 1500 and 2000 mEq/; however, the main part of the salivary sample collected at T_0_ revealed a higher value. These data could be related to the age of the patients: puberty is in fact characterized by important hormonal changes related to development [38] and by an increase in cellular metabolism related to the acceleration of somatic growth and sexual maturation. [36] The secondary purpose of this research was to investigate the influence of orthodontic multi-bracket therapy on oral hygiene and the consequent effect on oxidative stress. An experimental study on the influence of dental caries and hygiene on the amount of oxidant agents [38] stated that oxidative stress in patients with insufficient oral hygiene and dental caries is higher than in patients without plaque and tooth decay. Even if multi-bracket orthodontic appliances, like other type od prosthetic restoration [39-41], usually produce an increase in dental plaque, in the present study, we observed an improvement in dental hygiene during orthodontic therapy; these data could be related to the correct instruction and the continuous monitoring of the patients enrolled in the study [42-43]. The study did not reveal any correlation between the level of dental hygiene and that of oxidative stress.

## CONCLUSION

In conclusion, it is possible to state that orthodontic multi-bracket therapy did not influence the oxidative stress in the oral cavity during the first 10 weeks of treatment; these data could also be related to the use of a self-ligating vestibular appliance, which reduces the forces exerted on the teeth and allows for better dental hygiene during orthodontic treatment.

## Figures and Tables

**Fig. (1):**
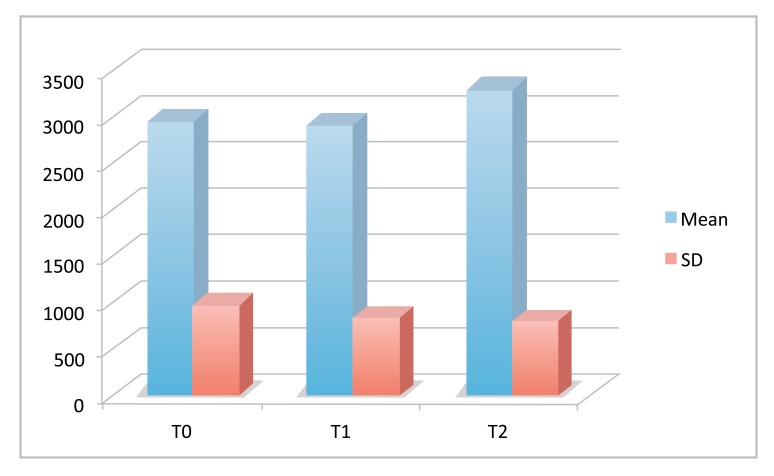
SAT Test results at the different time points.

**Fig. (2):**
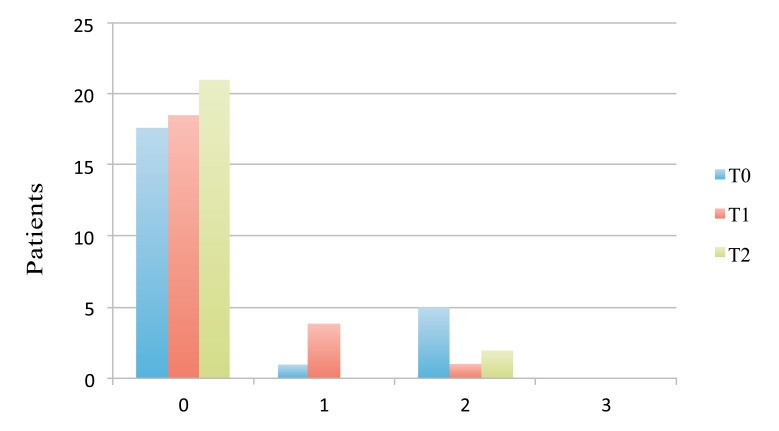
Distribution of plaque index scores at the different time points.

**Table 1:** Descriptive statistic (mEq/l).

**Timing**	**Mean**	**SD**	**Min**	**Mdn**	**Max**
Before Bonding	2954	974	1498	2986	4183
5 weeks	2903	795	1599	2835	4026
10 weeks	3297	798	2063	3189	4887

**Table 2:** Distribution of plaque index value in patients at the different time point.

**Score**	**Before Bond.**	**5 Weeks**	**10 Weeks**
0	17	18	21
1	1	4	0
2	5	1	2
3	0	0	0

**Table 3:** Correlation between plaque index scores and SAT results.

**T0**		
	Frequency (n°)	Mean (mEq/l)
PI 0	17	2960
PI 1	1	3022
PI 2	5	2920
TOTAL	23	2954
**T1**		
	Frequency (n°)	Mean (mEq/l)
PI 0	18	2897
PI 1	4	2952
PI 2	1	2815
TOTAL	23	2903
**T2**		
	Frequency (n°)	Mean (mEq/l)
PI 0	21	3301
PI 1	0	ND
PI 2	2	3255
TOTAL	23	3297
